# Impact of suboptimal dosimetric coverage of pretherapeutic 18F-FDG PET/CT hotspots on outcome in patients with locally advanced cervical cancer treated with chemoradiotherapy followed by brachytherapy

**DOI:** 10.1016/j.ctro.2020.05.004

**Published:** 2020-05-11

**Authors:** François Lucia, Vincent Bourbonne, Dorothy Gujral, Gurvan Dissaux, Omar Miranda, Maelle Mauguen, Olivier Pradier, Ronan Abgral, Ulrike Schick

**Affiliations:** a Radiation Oncology Department, University Hospital, Brest, France; b Nuclear Medicine Department, University Hospital, Brest, France; c Clinical Oncology Department, Imperial College Healthcare NHS Trust, Charing Cross Hospital, Hammersmith, London, UK; d Department of Cancer and Surgery, Imperial College London, London, UK

**Keywords:** cervical cancer, Image-guided, Brachytherapy, PET/TDM, Hotspot

## Abstract

**Introduction:**

Areas of high uptake on pre-treatment ^18^F-fluorodeoxyglucose (^18^F-FDG) positron emission tomography/computed tomography (PET/CT), denoted as “hotspots”, have been identified as preferential sites of local relapse in locally advanced cervical cancer (LACC). The purpose of this study was to analyze the dosimetric coverage of these hotspots with high dose-rate brachytherapy (BT).

**Methods:**

For each patient, a rigid registration of the CT from the pre-treatment PET/CT with the radiotherapy planning CT was performed using 3D Slicer^TM^, followed by a manual volume correction by translation and deformation if necessary. The fuzzy locally adaptive Bayesian (FLAB) algorithm was applied to PET images to simultaneously define an overall tumour volume and the high-uptake sub-volume V1. The inclusion of V1 in the high-risk clinical target volume (CTV HR) and its dosimetric coverage were evaluated using 3D Slicer^TM^. The average of the 3–4 BT sessions was reported.

**Results:**

Forty-two patients with recurrence after chemoradiotherapy (CRT) for LACC were matched to 42 patients without recurrence. Mean ± standard deviation follow-up was 26 ± 11 months. In the recurrence group, V1 was not included in the CTV HR and not covered by the 85 Gy isodose in 17/42 patients (41%) (1/20 with pelvic recurrence and 16/22 with distant recurrence) and not by the 80 Gy isodose in 7/42 patients (17%) (all with distant recurrence). In the non-recurrence group, V1 was not included in CTV HR and not covered by the 85 Gy isodose in 3 patients only (7%). The hotspots coverage by the 85 Gy isodose was significantly better in patients who did not recur, but only when compared to patients with distant relapse (p < 0.0001).

**Conclusion:**

Suboptimal dosimetric coverage of high FDG uptakes on pretherapeutic PET could be associated with an increased risk of recurrence.

## Highlights

• Hotspots can be easily identified on the pretherapeutic PET in patients with cervical cancer.
• Registration of PET with planning CT allows for the dosimetric coverage evaluation of these hotspots.
• The initial hotspot was not entirely included in the CTV-high risk in 40% of patients who recur during the follow-up, compared to 7% in patients without recurrence.
• Hotspot was not entirely included in the CTV-high risk in 40% of patients who recur-Hotspots-guided radiotherapy could be applied easily in daily routine.

## 1 Introduction

Cervical cancer (CC) is the third most common malignancy and the fourth most common cause of cancer-related deaths in women worldwide [1]. Seventy to eighty percents of patients are diagnosed with locally advanced disease [2]. In these patients, the standard of care consists of pelvic external beam radiotherapy (EBRT) in combination with cisplatin-based chemotherapy, and subsequent brachytherapy (BT). However, despite recent advances in cervical cancer management especially in image-guided radiotherapy and image-guided brachytherapy, approximately 40% of patients present a recurrence after curative intent treatment, and eventually die of disease [2].

18F-fluorodeoxyglucose (18F-FDG) positron emission tomography/computed tomography (PET/CT) is recommended for initial staging [3] or at the time of recurrence of CC and could also have an interesting role in evaluating response to treatment [3], [4]. In addition, the intensity of 18F-FDG uptake is a valuable prognostic factor: pre-treatment maximum standardized uptake value (SUVmax) has been shown to predict the presence of lymph nodes metastasis at diagnosis, and an independent predictor of recurrence and survival [5]. For brachytherapy, a residual gross tumor volume (GTV), a high-risk clinical target volume (CTV-HR) and an intermediate-risk clinical target volume (CTV-IR) are defined according to (i) gynecological examination and (ii) MRI at diagnosis and at the time of brachytherapy [6]. However, the role of PET in radiotherapy planning and particularly in volume delineation has been poorly studied in patients with CC [7], [8], [9], [10], [11], [12].

Pre-treatment high 18F-FDG uptake areas on PET/CT denoted as “hotspots” have been identified as preferential sites of local relapse after chemoradiotherapy (CRT) in many tumor subtypes including non-small cell lung cancer [13], rectal cancer [14], head and neck cancer [15] and esophageal cancer [16]. We previously found that there is a good correlation between the site of recurrent disease and the more active intratumoral region on the baseline PET/CT in locally advanced CC [17].

The identification of these hotspots on the staging PET/CT could guide the dose distribution of the BT plan and improve outcome whilst minimizing irradiation of surrounding tissues. We therefore investigated the dosimetric coverage of these hotspots during the brachytherapy procedure, in patients with or without recurrent disease.

## 2 Materials and Methods

### 2.1 Patients

All patients with histologically proven locally advanced CC, staged IB1-IVA (FIGO 2009 definition [18], (The classification of patients according to FIGO 2018 staging is available in table 1, supplementary material) and treated at our institution with definitive curative CRT and subsequent BT from September 2012 to December 2017 and who developed recurrence during follow-up were included in this retrospective study. A control group of patients with the same clinical- (age, tumor volume, FIGO staging, lymph nodes status) and treatment- (type of EBRT, BT dose, chemotherapy) -related characteristics who did not develop any recurrence during follow-up was also identified for the purpose of matching. A minimum follow-up of 6 months was mandatory. Patients with a history of previous chemotherapy or RT and/or metastatic disease were excluded. None of the patients received adjuvant chemotherapy. All patients underwent an initial pre-treatment 18F-FDG PET/CT as part of the initial staging (PET1) and at the time of recurrence.

**Table 1:** Patients’ characteristics.

	Recurrence		No recurrence		p
	n = 42	%	N = 42	%	
Age median (range)	54 (32–79)		53 (30–78)		0.88
FIGO stage					
IB1	2	5	2	5	0.62
IB2	2	5	2	5	0.62
IIA	2	5	2	5	0.62
IIB	24	57	24	57	0.83
IIIA	2	5	2	5	0.62
IIIB	6	14	6	14	0.75
IVA	4	9	4	9	0.70
Histology					
Squamous carcinoma	34	81	34	81	0.78
Adenocarcinoma	6	14	6	14	0.75
Adenosquamous carcinoma	0	0	0	0	
Clear cell carcinoma	2	5	2	5	0.62
Grade					
I	16	38	16	38	0.82
II	16	38	17	41	0.95
III	10	24	9	21	0.95
Lymph node involvement					
Uninvoled	18	43	18	43	0.83
Involved	24	57	24	57	0.83
pelvic	16	67	16	67	0.76
pelvic and *para*-aortic	8	33	8	33	0.76
V1 (mean ± STD)	14.8 ± 10.6 cm^3^		14.4 ± 10.2 cm^3^		0.86
MTV (mean ± STD)	42.6 ± 32.1 cm^3^		38.0 ± 22.9 cm^3^		0.45
SUV_max_ (mean ± STD)	19.9 ± 8.1		19.4 ± 7.5		0.77
Treatment					
3D-CRT	30	71	30	71	0.81
IMRT	12	29	12	29	0.81
EBRT dose median (range)	45 (45–54)		45 (45–54)		1.00
BT dose median (range)	24 (21–26)		24 (21–26)		1.00
D98 GTV res median (range)	99.6 (93.1–102.5)		99.5 (92.8–102.3)		0.95

Abbreviations: FIGO = International Federation of Gynecology and Obstetrics, V1: high-uptake sub-volumes, MTV: metabolic tumor volume, TLG: total lesion glycolysis, 3D-RT = three-dimensional conformal radiotherapy, IMRT = intensity-modulated photon radiotherapy, EBRT = external beam radiotherapy, BT = brachytherapy, D98 GTV res: dose of 98Gy_a/b = 10_ to the Residual Gross Tumour Volume of the primary Tumour.

Collected data included age and date of diagnosis, histology, FIGO stage, presence of positive lymph nodes on 18F-FDG PET/CT, tumor size as measured on MRI, EBRT and BT dose, date and site of recurrence, as well as date and status at last follow-up. Recurrences were considered as local (vaginal and/or cervical), regional (pelvic/*para*-aortic), or distant (upper abdominal and/or extra-abdominal) [19].

All patients provided signed informed consent for the use of their clinical data for scientific purposes and for the anonymous publication of data. Our Institutional Review Board approved this study (29BRC18.0015).

### 2.2 Imaging

#### 2.2.1 PET/CT acquisition

Scans were performed on a Biograph mCT S64™ (Siemens® Healthineers Medical Solutions, Knoxville, TN, United States) for all patients. Standard preparation included at least 4 h of fasting and a serum blood glucose level <7 mmol/L before tracer administration. PET acquisitions were carried out approximately 60 min after injection of 3 MBq/kg of 18F-FDG.

The Biograph scanner consisted of a 64-slice multidetector-row spiral CT with a transverse field of view of 700 mm. Standard CT parameters were used: collimation of 16 × 1.2 mm^2^, pitch 1, tube voltage of 120 kV, and effective tube current of 80 mAs. 3D PET data were reconstructed using an ordered subsets expectation–maximization (OSEM) algorithm (true X 5 point spread function + time of flight OSEM-three dimensional [3D]).

### 2.3 Treatment

Consortium guidelines were applied to outline the GTV, the CTV, the planning target volume (PTV) and organs-at-risk [20]. Treatment consisted of three-dimensional conformal radiotherapy (3DRT) (n = 30 in each group) or intensity-modulated radiotherapy (IMRT) (n = 12 in each group) delivered using a linear accelerator (ONCOR™ Digital Medical Linear Accelerator from Siemens® Medical Solutions, Inc. or a TrueBeam STx Novalis Linear Accelerator), followed by high-dose-rate (HDR) intracavitary BT.

All patients received pelvic EBRT or extended-field RT to the *para*-aortic area using high energy photons (18 MV), at a dose of 45 Gy using standard fractionation. For patients with positive pelvic or *para*-aortic lymph nodes, an image-guided targeted boost was delivered sequentially up to a total dose of 54 Gy to the involved nodes. However, for 6 patients (3 in each group) a dose of 50.4 Gy only was delivered due to OAR constraints (Table 2, supplementary material).

**Table 2:** PET tumor characteristics.

	Recurrence		No recurrence	
	pelvic	distant	pelvic	distant
V1 (mean ± STD)	18.2 ± 13.5 cm^3^	11.7 ± 7.8 cm^3^	16.6 ± 12.5 cm^3^	12.7 ± 11.4 cm^3^
MTV (mean ± STD)	41.9 ± 31.6 cm^3^	43.5 ± 32.9 cm^3^	36.3 ± 21.9 cm^3^	39.6 ± 24.0 cm^3^
SUV_max_ (mean ± STD)	19.5 ± 7.4	20.2 ± 8.7	19.7 ± 7.8	19.1 ± 7.1
SUV_mean_ (mean ± STD)	6.6 ± 2.9	8.1 ± 4.5	6.8 ± 3.0	7.0 ± 3.4
TLG (mean ± STD)	286.4 ± 281.5 g	301.4 ± 287.2 g	287.3 ± 275.4 g	292.7 ± 269.5 g

Abbreviations: V1: high-uptake sub-volumes, MTV: metabolic tumor volume, SUV_max_: maximum standardized uptake value, SUV_mean_: mean standardized uptake value, TLG: total lesion glycolysis.

Patients received 3–4 fractions of MRI-guided HDR intracavitary BT every 4 days (Table 2, supplementary material), which commenced one week after EBRT completion. The prescribed dose was 6–7 Gy to the high-risk CTV. The following dose constraints were applied: CTV-HR D90 (EQD2_10_) ≥ 85 Gy, CTV-IR D90 (EQD2_10_) ≥ 65 Gy, GTV D98 (EQD2_10_) ≥ 90 Gy, D2cm^3^ of bladder <90 Gy, D2 cm^3^ of rectum <75 Gy, and D2 cm^3^ of sigmoid/bowel <75 Gy [6]. The reference ICRU (International Commission of Radiation Units) recto-vaginal point dose had to receive less than 75 Gy. A new plan was performed for each brachytherapy fraction. The delineation of the volumes of interest was performed on the planning CT with help of the MRI.

All patients received 4–6 cycles of concomitant chemotherapy with weekly cisplatin (40 mg/m^2^) or carboplatin (AUC 2) in case of renal contraindication (Table 2, supplementary material).

### 2.4 Follow-up

Clinical follow-up consisted of physical examination every three months until 2 years after diagnosis, every 6 months up to 5 years, annually thereafter, and was done alternatively by the radiation oncologist and gynaecologist. Follow-up imaging studies consisted of MRI and 18F-FDG PET/CT at 3 months after treatment and annually until 2 years after treatment completion, CT every 6 months until 2 years after treatment completion and if clinically indicated thereafter, and/or 18F-FDG PET/CT if clinically indicated.

### 2.5 Overlap

#### 2.5.1 PET/CT and CT planning registrations

For each patient, a rigid registration of the CT component of the pretherapeutic PET/CT with the radiation planning CT was performed using the 3D Slicer TM Expert Automated Registration module [21] optimized with the Mattes mutual information metric [22]. The transform was initialized with a registration of the two centers of mass of the images with a box centered on the cervix. The obtained transform was then applied to the corresponding PET. In cases of obvious misalignments (in case of tumor response and/or deformation by the applicator at the time of BT), manual adjustments by translation were allowed.

#### 2.5.2 Volumes determination

We exploited PET images only. The fuzzy locally adaptive Bayesian (FLAB) algorithm previously validated for automatic tumor volume delineation [23], [24] was used. Indeed, in the absence of ground-truth and based on previous results, FLAB was assumed to provide more accurate and robust volumes compared to fixed thresholds [25], [26]. FLAB was applied using 3 classes (one for background and the other two for tumor) to simultaneously define an overall tumor volume and the high-uptake sub-volume, referred to V1 [27].

#### 2.5.3 Coverage analysis

First, we converted the CTV-HR into a volume and then evaluated the inclusion of the segmented high-uptake sub-volumes (V1) in the CTV-HR for each brachytherapy session. This was done using the “Dose Volume Histogram” module in 3D Slicer. The average of the 3–4 BT sessions was reported. We also evaluated the coverage of the V1 by different isodose lines (from the 85 Gy isodose line to the isodose that allowed 100% coverage of the CTV-HR).

To evaluate the spatial distribution of the hotspots, all PET sets with delineated hotpots were registered with a reference planning scan from a standard patient after automatic deformable registration using the ATLAS option of the MIM Vista® 6.5.2 Software.

### 2.6 Statistical analysis

Patients in the recurrence cohort were individually matched to patients in the non recurrence cohort. Matching criteria used were tumor volume, stage (FIGO IB1 to IVA), lymph nodes involvement and age. Where an exact match according to the above criteria was not possible, the method of minimization was used to restrict differences between patients. In cases of more than one exact match to a “recurrence patient”, one “non recurrence patient” was randomly selected from all possible exact matches. When patients with local relapse or distant relapse specifically were analyzed, the corresponding subgroup of patients was considered.

## 3 Results

### 3.1 Patient and tumor characteristics

**Fig. 1:**
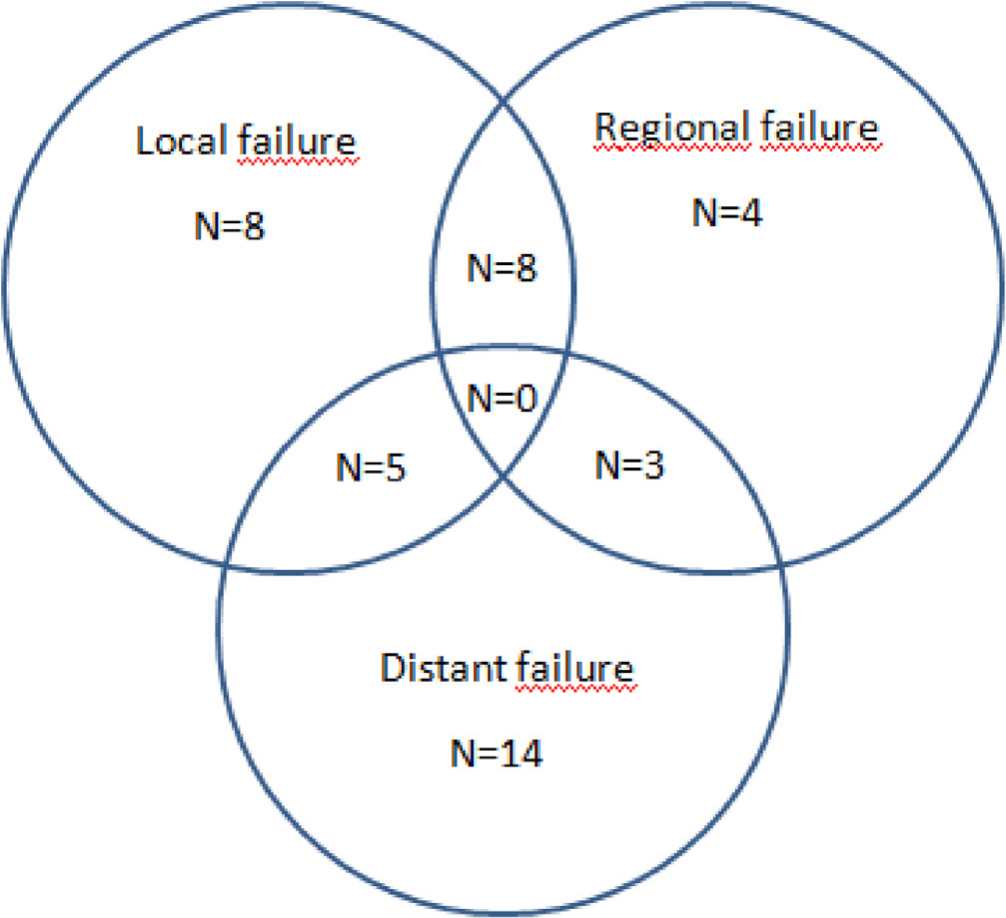
Venn diagram of failure.

Eighty four patients were included, forty-two in each group. Patient characteristics are shown in Table 1. The mean ± SD follow-up was 26 ± 11 months. No patient experienced delays or breaks in EBRT due to short-term toxicity (median RT duration, 49 days; range, 47–51 days). Among the 42 patients with recurrence, 20 patients were still alive and 22 had died from the disease at the time of analysis. Eight patients had an isolated local recurrence, 8 had local and nodal recurrences, 5 had local and distant recurrences, 4 had an isolated regional recurrence, 3 had regional and distant recurrences, and 14 had an isolated distant recurrence (Fig. 1). As new biopsies were not performed in the 4 patients having isolated regional recurrence and in 6 patients having distant recurrence, pathological confirmation of recurrence was available in only 32 patients (76.2%).

### 3.2 Registration procedure and tumor volumes

Amongst patients who recurred during the follow-up, a manual correction by translation and deformation after rigid registration was required in 28 women (10 pelvic recurrences/18 distant recurrences) due to significant anatomical variations of bladder and/or rectum filling, tumor response and/or deformation by the applicator at time of BT. According to FLAB, the mean entire tumor volume on PET was 42.6 ± 32.3 cm^3^ and was significantly larger than the mean high-uptake sub-volumes (V1) of 15.3 ± 11.8 cm^3^ (p < 0.01).

Mean SUVmax, SUVmean and total lesion glycolysis (TLG) values were 19.8 ± 7.9, 7.1 ± 3.7 and 294.5 ± 278.3 g.

The PET tumor characteristics in women with either pelvic or distant relapse are presented in Table 2.

For patients without recurrences, a manual correction was required in 27 patients by translation and deformation after rigid registration due to significant anatomical variations in bladder and/or rectum filling, tumor response and deformation by the applicator at time of brachytherapy.

According to FLAB, the mean entire tumor volume was 38.0 ± 22.9 cm^3^ and was significantly larger than the mean high-uptake sub-volumes (V1) of 14.4 ± 10.2 cm^3^ (p < 0.01).

Mean SUVmax, SUV mean and TLG values were 19.4 ± 7.5, 6.9 ± 3.2 and 289.3 ± 271.2 g on PET1.

The PET tumors characteristic of these patients matched with patients with pelvic and with distant relapse are presented in Table 2.

### 3.3 Coverage of the initial high-uptake sub-volume (V1) by CTV-HR

**Fig. 2:**
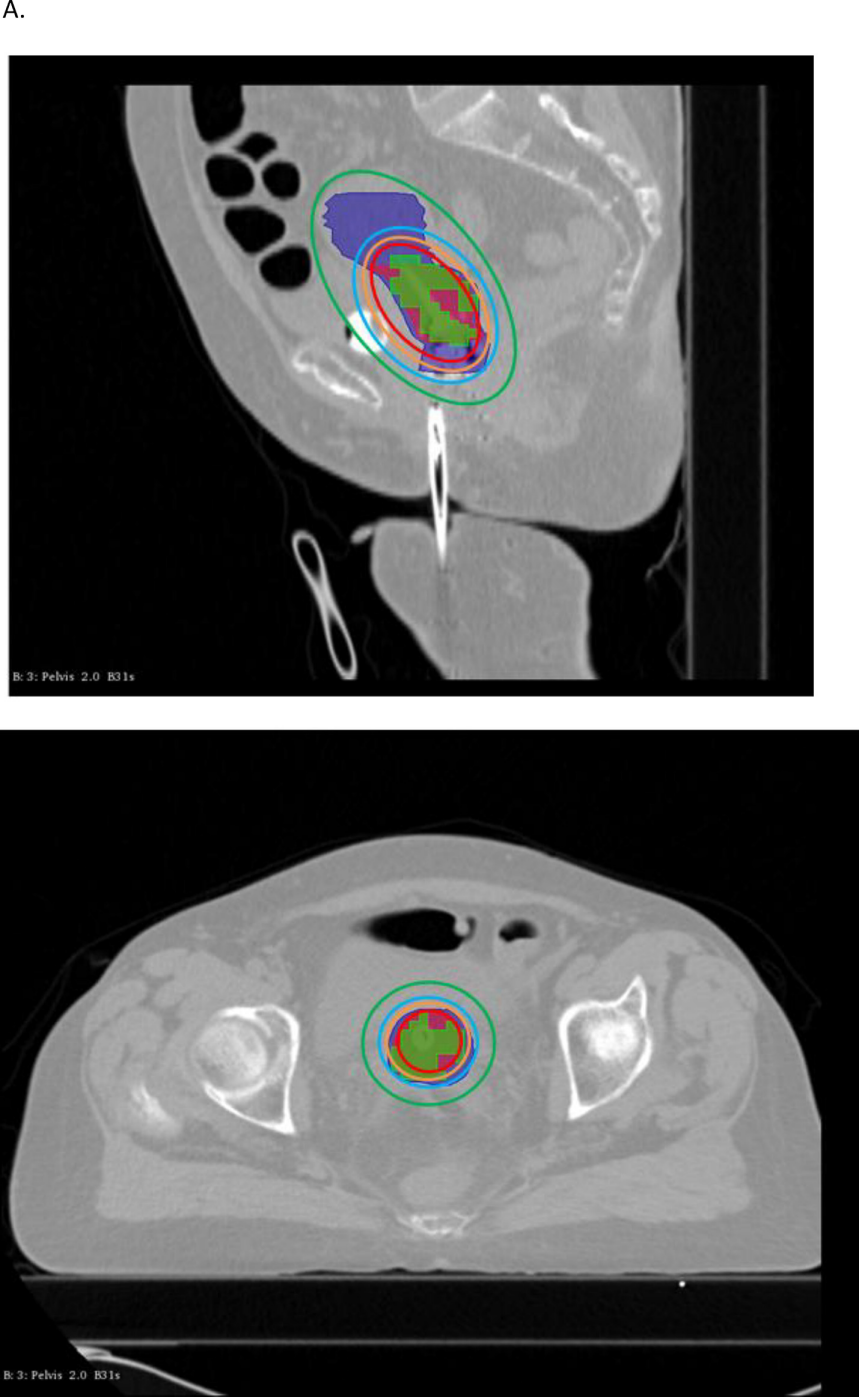

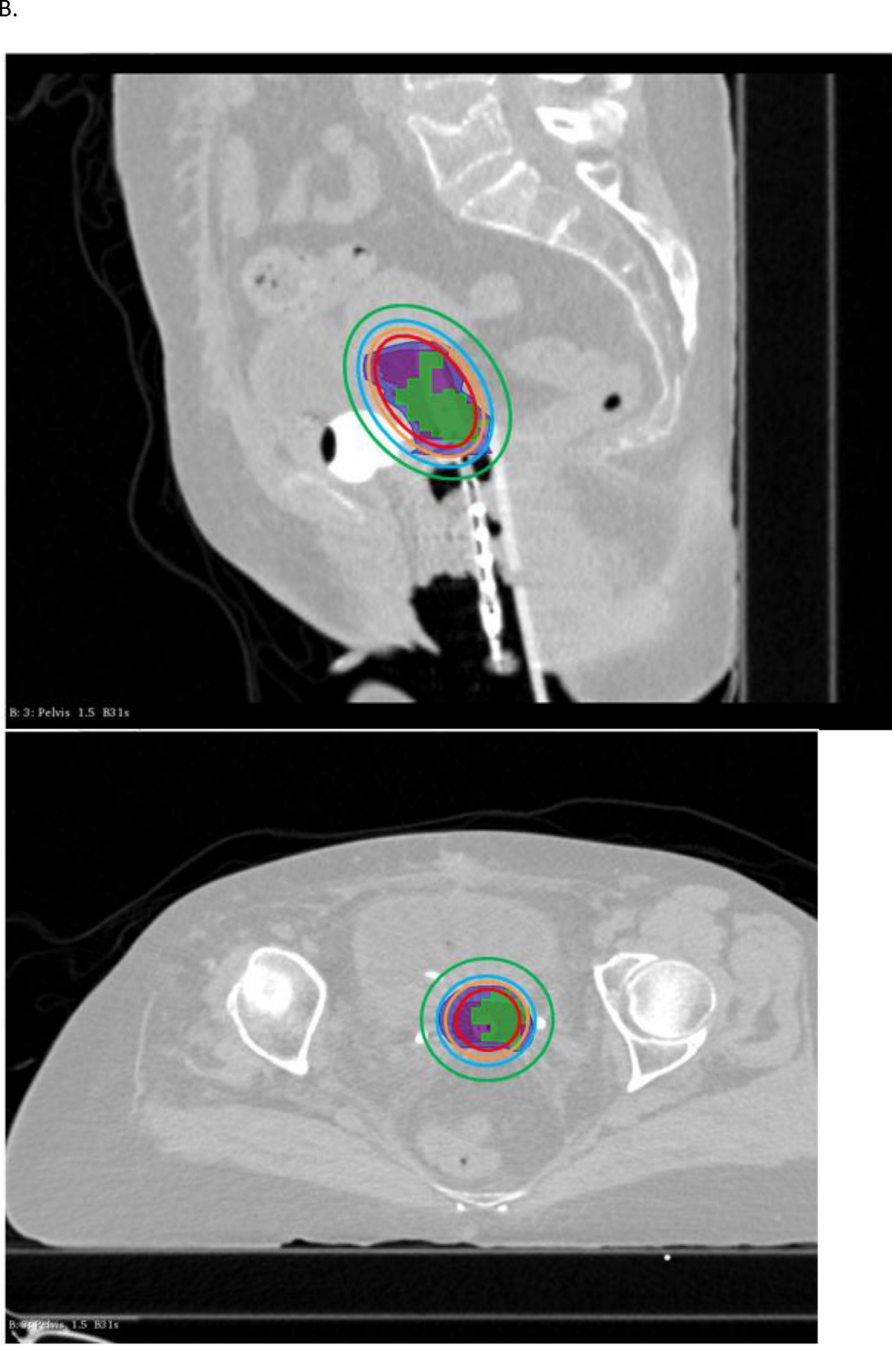
Examples of brachytherapy planning in 2 different patients who experienced distant relapse. In these 2 cases, the entire V1 (green) was not included in the CTV-HR (magenta volume) and not covered by the D_2eq_ = 85 Gy (red), neither by the 80 Gy isodose (orange). It was however included in the CTV IR (blue volume) and covered by the 78 Gy (cyan) and 65 Gy isodoses (green). (For interpretation of the references to colour in this figure legend, the reader is referred to the web version of this article.)

**Fig. 3:**
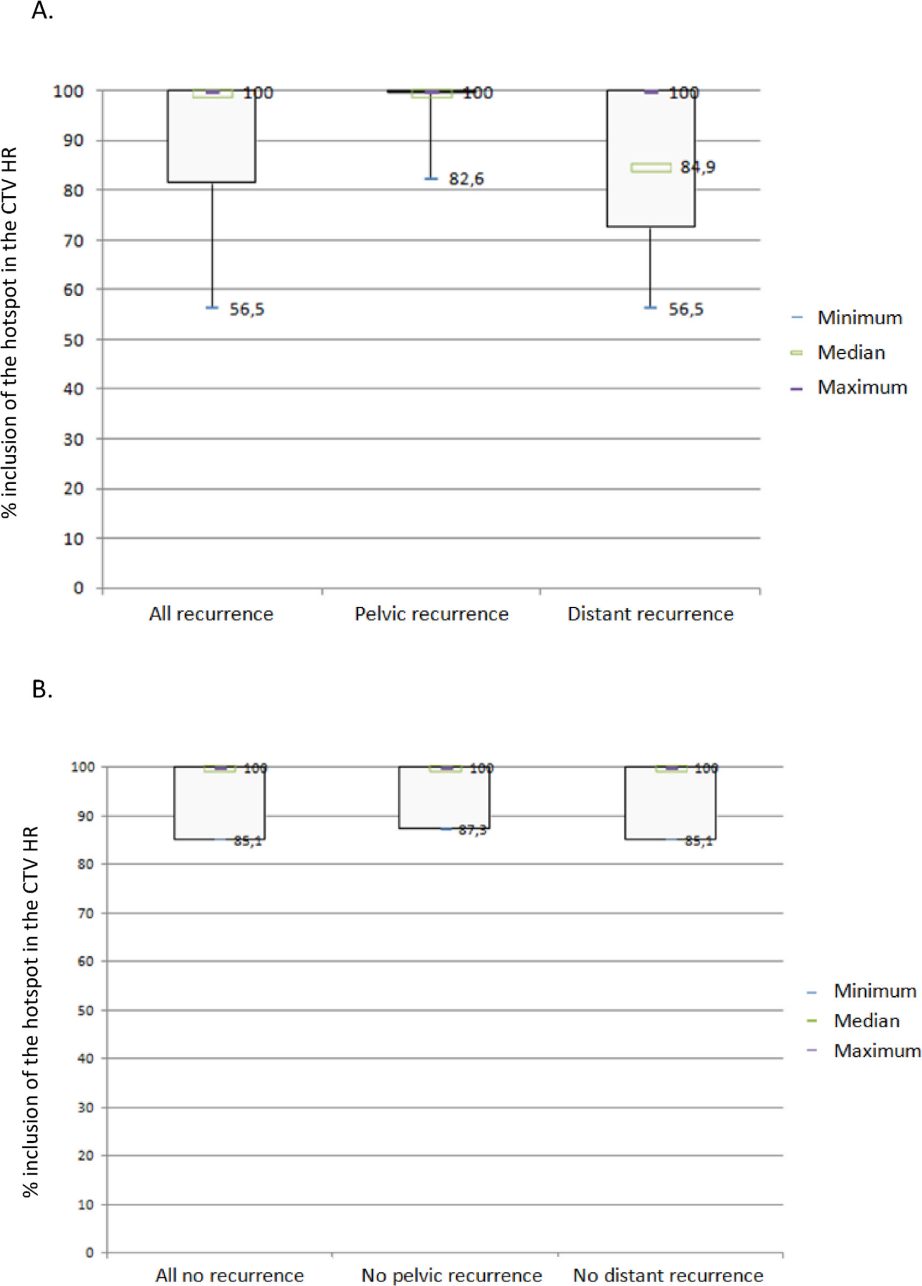
Box plots representing the inclusion of the hotspot in the CTV HR (A) in the recurrence group and (B) in the non recurrence group, with a significant difference observed between patients with and without distant recurrence (p < 0.0001), but without difference between the other subgroups.

When considering patients who relapsed, V1 was not included in the CTV-HR in 40.5% of patients and not covered by the 85 Gy, neither by the 80 Gy isodose in 17/42 patients (40.5%) and 7/42 patients (16.7%), respectively (examples in Fig. 2). The mean inclusion of V1 in CTV-HR was 90.3 ± 13.9% (Fig. 3) and the coverage of V1 by the 85 Gy (Fig. s1A, supplementary material), 80 Gy (Fig. s2A, supplementary material), and 78 Gy (Fig. s3A, supplementary material) isodoses were 92.1 ± 12.1%, 98.4 ± 4.0% and 100%, respectively.

For patients in the non recurrence group, V1 was not included in the CTV-HR and not covered by the 85 Gy isodose in 7.1% patients. The mean inclusion of V1 in the CTV-HR was 99.1 ± 3.4% (Fig. 3) and the mean coverage of V1 by the 85 Gy (Fig. s1B, supplementary material), 80 Gy (Fig. s2B, supplementary material), and 78 Gy (Fig. s3B, supplementary material) isodoses were 99.4 ± 2.2%, 100% and 100%, respectively. There was a significant difference between inclusion in the CTV HR and coverage by isodose 85 Gy in patients without recurrence compared to patients with distant recurrence (p < 0.0001) (Fig. 3).

When considering patients with pelvic recurrence specifically, V1 was not entirely included in CTV-HR and not covered by the 85 Gy isodose in one only patient, and this rate was similar in the matched patients who did not recur locally (5%). On average, CTV-HR included 99.1 ± 3.9% of V1 (Fig. 3) and the V1 coverages by the 85 Gy (Fig. s1A, supplementary material), 80 Gy (Fig. s2A, supplementary material), and 78 Gy (Fig. s3A, supplementary material) isodoses were 99.4 ± 2.9%, 100% and 100%, respectively, compared to 99.4 ± 2.8% (p = 0.51) and 99.6 ± 1.7% (p = 0.42), 100% (p = 1.00) and 100% (p = 1.00) in the matched group. There was no significant difference between the 2 groups.

In patients who developed distant relapse, V1 was not entirely encompassed in the CTV-HR nor covered by the 85 Gy isodose in 16/22 (72.7%) patients, compared to 9.1% in the matched group (p < 0.0001). In 7/22 patients (31.8%), V1 was not covered by the 80 Gy isodose, whereas it wasn’t the case for any of the matched patients (p < 0.0001). The mean inclusion of V1 in the CTV-HR was 83.1 ± 15.1%; this was significantly lower than in the non distant recurrence group (98.7 ± 4.0%) (p < 0.0001) (Fig. 3). The V1 coverage by the 85 Gy (Fig. s1B, supplementary material), 80 Gy (Fig. s2B, supplementary material), and 78 Gy (Fig. s3B, supplementary material) isodoses were 86.1 ± 13.5%, 97.1 ± 5.1% and 100% versus 99.2 ± 2.2% (p < 0.0001), 100% and 100% in the non distant recurrence group.

All patients had 100% coverage of V1 by CTV-IR.

**Fig. 4:**
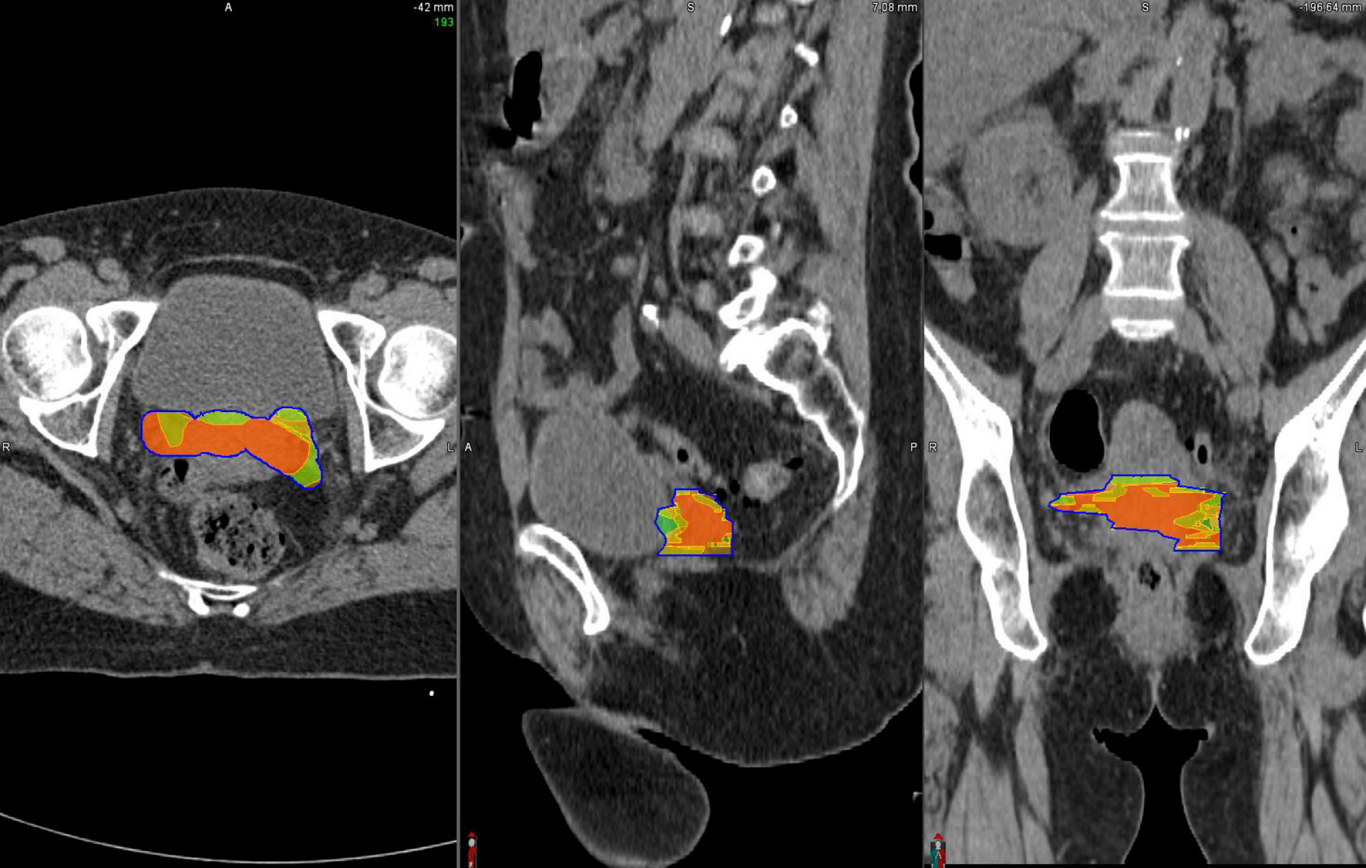
Spatial distribution of all hotspots obtained after deformable registration of the PET imaging sets with a single planning CT. Volumes in red representing the presence of > 75% hotspots, orange 50–75%, yellow 25–50% and green < 25%. (For interpretation of the references to colour in this figure legend, the reader is referred to the web version of this article.)

The spatial distribution of the different hotspots is illustrated in Fig. 4.

## 4 Discussion

The use of 18F-FDG PET/CT has already become a standard for radiotherapy planning and target volumes outlining in some tumors such as lymphoma or lung cancer [28]. However, in patients with cervical cancers PET/CT is only currently used for detection of lymph node metastases. To our knowledge, this is the first study showing that FDG hotspots on baseline 18F-FDG PET/CT could guide the CTV delineation during BT in locally advanced CC.

Our study showed that the dosimetric coverage of the initial high-uptake PET sub-volume by the 80% isodose was not achieved in more than 40% of patients who relapsed during follow-up, and dosimetric coverage of this subregion was significantly lower compared to patients without recurrence. Therefore, we recommend including these areas in the CTV HR to ensure adequate coverage during planning.

Surprisingly, patients with suboptimal coverage of hotspots seemed to develop more distant recurrence compared to patients with better coverage, when we would have expected more local failures. Insufficient dose may allow growth of radioresistant tumor clones responsible for subsequent metastatic recurrence [29]. Moreover, we can’t exclude that microscopic local recurrences were initially associated with distant recurrences in these patients, but not visible on imaging because of the action of systemic treatments.

A strong dose-effect relationship for local control exists in the treatment of CC: in these two series of 156 and 592 patients, dose to the HR-CTV of at least 86 Gy and 92 Gy were associated with local control rates higher than 90% and 95%, respectively [30], [31].

However, recently, the patterns of failure from the RetroEMBRACE Study showed that, with the use of image-guided adaptive brachytherapy, the patterns of relapse after chemoradiation have changed, with the predominant failure being systemic, whereas the predominant failure with conventional brachytherapy was pelvic [32]. Our findings are thus particularly interesting in this context, and in line with another study reported by Chargari et al. in 109 patients treated for a LACC by CRT and BT. This study showed that a lower ability to reach the target D90 to the HR-CTV planning and an HR-CTV volume ≥ 40 cm^3^ lead to a high propensity of distant relapse [33].

Some studies have already investigated the predictive value of PET in addition to detecting lymph node involvement and metastasis. A *meta*-analysis of 12 studies highlighted the prognostic value of volume-based 18F-FDG PET/CT parameters (metabolic tumor volume (MTV) and TLG) [34] in 660 patients, although conflicting results were reported by another study [35]. A study including 50 patients evaluated MRI- and PET-guided BT in patients with vaginal recurrence of CC after surgery [36] and reported that MTV > 15 cc (using 35% of the SUVmax by the gradient tumor segmentation method) resulted in inferior local control and a trend towards poorer overall survival. However, these studies only evaluated the prognostic value of MTV and did not propose how MTV could be incorporated in the delineation process. Another report on 29 patients evaluated the concordance of the anatomical tumour volume defined on T2-weighted MRI (considered as the gold standard) with the MTV measured on 18F-FDG PET/CT [37]. A very good correlation was found between MTV30 (30% threshold of SUVmax) and anatomical tumor volume. Despite the low number of patients, these results are interesting and support the use of functional 18F-FDG PET/CT imaging as a surrogate of MRI for radiotherapy and image-guided brachytherapy planning. The incorporation of functional imaging could go further than simple target definition, as PET may help to identify hypermetabolic sub-volumes and guide dose- painting in LACC.

Our study has limitations. Firstly, that is a monocentric and retrospective series, with a small number of patients. Additionally, only primary tumors were analyzed. Moreover, despite the use of a validated automatic registration method, manual correction was required in some patients due to significant anatomical variations related to variations in bladder and/or rectum repletion. These corrections can lead to intra- or inter-observer variabilities. Finally, our findings are based on the hypothesis that V1 locations are not influenced by tumor shrinkage. Indeed, V1 are extracted from the pretherapeutic PET whereas CTV HR is delineated on the planning CT (with help of MRI) performed after EBRT, which usually allows a tumor volume decrease of 50% [38]. This could possibly over/underestimate the amount of V1 inclusion in the CTV HR.

Despite these potential sources of bias, identifying 18F-FDG hotspots on initial 18F-FDG PET/CT is a promising approach for personalized treatment in patients undergoing CRT with inherent limitations that will need to be addressed before it can be used in clinical practice. Amongst these, the repeatability and robustness of the procedure has to be improved.

Accurate segmentation is the first important step in identifying 18F-FDG hotspots. This is, however, straightforward, taking approximately one minute with FLAB. Although FLAB is not freely available, other efficient PET segmentation tools are available in clinical practice, such as adaptive thresholding or gradient-based method [25]. Therefore, our results should be reproducible by others. The registration between the planning CT and the CT component of the PET/CT is also a crucial step which requires 2–3 min, but does not require additional tools. Indeed, radiation oncologists deal now with functional and molecular imaging to increase the definition of target volumes in daily practice, and are increasingly familiar with registration between various imaging modalities, especially with the emergence of stereotactic radiotherapy [39], [40]. To accommodate this, radiation oncology planning softwares now provide registration functionalities. The registration parameters are then easily and rapidly applied to the PET. Finally, hotspot outlining is exported to the brachytherapy software and its inclusion in the CTV HR is evaluated (one minute step). In total, we calculated a workload of less than 10 min is required for the entire procedure, which is certainly achievable in daily clinical practice.

Our previous results suggested that 2 radiomics features in 18F-FDG PET and in ADC maps from Diffusion-weighted MRI (DWI MRI) are powerful predictors of the efficacy of CRT in the treatment of CC [35]. Higher values of these parameters are associated with worse outcome, confirming that more heterogeneous tumors have a poor prognosis. These findings can be acted upon to tailor treatment.

Further work could analyse the correlation of the different radiomics features with V1. Two other aspects could also be investigated inside clinical trials, namely (i) BT dose escalation to pre-therapeutic identified hotpots in patients at high risk of isolated loco-regional relapse and (ii) a better coverage of the initial hotspot by the CTV HR in patients at high risk of metastatic relapse. Given the distribution of hotspots which are in part located within the parameters (Fig. 4), it is possible the use of interstitial brachytherapy would improve the hotpots dosimetric coverage, especially in patients with parametrial involvement or large tumors [6], [41].

These aspects have already been investigated in other tumor sites such as prostate cancer. Indeed, MRI has been studied to boost the predominant intra prostatic lesion (DIL) [42], [43] whereas the value of prostate-specific membrane antigen (PSMA) PET/CT has been investigated in a planning study to allow a boost on the DIL in external radiotherapy [44], [45].

## 5 Conclusion

Suboptimal dosimetric coverage of areas of high FDG uptake on pretherapeutic PET could be associated with an increased risk of CC recurrence. The identification of these hotspots on PET could guide the BT procedure in patients with CC. Further large prospective studies are needed to confirm and externally validate these observations.
