# Supplementary material for: Impact of suboptimal dosimetric coverage of pretherapeutic 18F-FDG PET/CT hotspots on outcome in patients with locally advanced cervical cancer treated with chemoradiotherapy followed by brachytherapy
Source: Clin Transl Radiat Oncol. 2020 May 11;23:50–9. doi: 10.1016/j.ctro.2020.05.004 (PMC7229342; doi:10.1016/j.ctro.2020.05.004)
Supplement: Supplementary data 1 [file mmc1.docx]

**Table 1: Staging according to FIGO 2018**

|  | Recurrence | | No recurrence | |
| --- | --- | --- | --- | --- |
|  | n=42 | % | N=42 | % |
| Age median (range) | 54 (32-79) |  | 53 (30-78) |  |
| FIGO stage |  |  |  |  |
| IB1 | 0 | 0 | 0 | 0 |
| IB2 | 2 | 5 | 2 | 5 |
| IB3 | 1 | 2 | 1 | 2 |
| IIA | 1 | 2 | 1 | 2 |
| IIB | 8 | 19 | 8 | 19 |
| IIIA | 0 | 0 | 0 | 0 |
| IIIB | 2 | 5 | 2 | 5 |
| IIIC1 | 16 | 38 | 16 | 38 |
| IIIC2 | 8 | 19 | 8 | 19 |
| IVA | 4 | 10 | 4 | 10 |

**Table 2: Treatments’ characteristics**

|  | | Recurrence | No recurrence | | p | | |
| --- | --- | --- | --- | --- | --- | --- | --- |
|  | n=42 | | % | N=42 | | % |  |
| External Beam Radiotherapy |  | |  |  | |  |  |
| 3D-CRT | 30 | | 71 | 30 | | 71 | 0.81 |
| IMRT | 12 | | 29 | 12 | | 29 | 0.81 |
| dose median (range) | 45 (45-54) | |  | 45 (45-54) | |  | 1.00 |
| Pelvic dose | 45 | |  | 45 | |  | 1.00 |
| Involved nodes dose | 54 (50.4-54) | |  | 54 (50.4-54) | |  | 1.00 |
| 50.4 | 3 | | 13 | 3 | | 13 | 0.67 |
| 54 | 21 | | 87 | 21 | | 87 | 0.67 |
| Chemotherapy  Cisplatin  Carboplatin  Number of cycles | 35  7 | | 83  17 | 36  6 | | 86  14 | 0.77  0.77 |
| 4 | 6 | | 14 | 5 | | 12 | 0.96 |
| 5 | 34 | | 81 | 35 | | 83 | 0.96 |
| 6 | 2 | | 5 | 2 | | 5 | 0.62 |
| Brachytherapy |  | |  |  | |  |  |
| dose median (range) | 24 (21-26) | |  | 24 (21-26) | |  | 1.00 |
| 21 (3 x 7 Gy) | 1 | | 2 | 1 | | 2 | 0.68 |
| 24 (4 x 6 Gy) | 6 | | 14 | 6 | | 14 | 0.96 |
| 26 (4 x 6.5 Gy) | 15 | | 36 | 15 | | 36 | 0.83 |
| 28 (4 x 7 Gy) | 20 | | 48 | 20 | | 48 | 0.85 |
| D98 GTV res median (range) | 99.6 (93.1-102.5) | |  | 99.5 (92.8-102.3) | |  | 0.95 |

Abbreviations: 3D-RT= three-dimensional conformal radiotherapy, IMRT= intensity-modulated photon radiotherapy, EBRT= external beam radiotherapy, BT=brachytherapy, D98 GTV res: dose of 98Gy_a/b = 10_ to the Residual Gross Tumour Volume of the primary Tumour

Figure s1. Box plots representing the coverage of the hotspot by isodose 85Gy (A) in the recurrence group and (B) in the non recurrence group , there was a significant difference for the subgroup who experienced distant recurrence vs who the one who did not non distant recurrence (p<0.0001).

A.


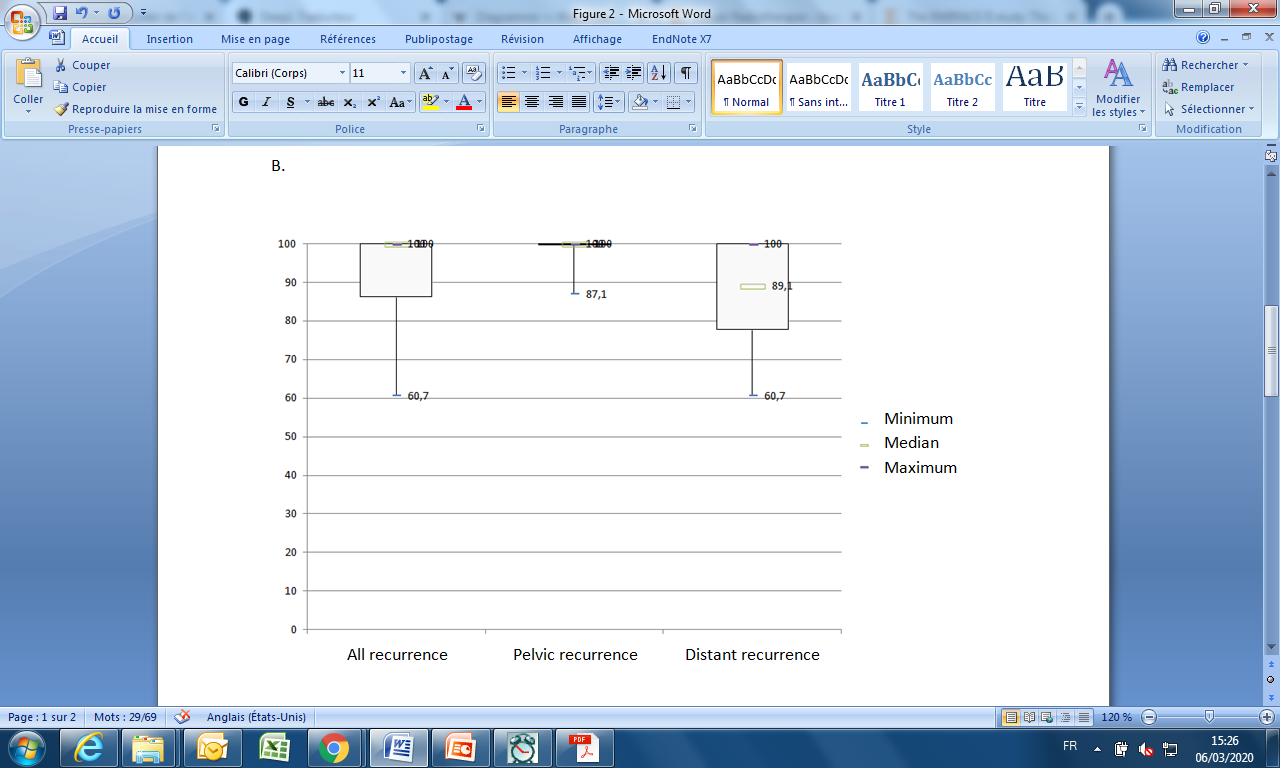


% coverage of the hotspot by isodose 85Gy

B.


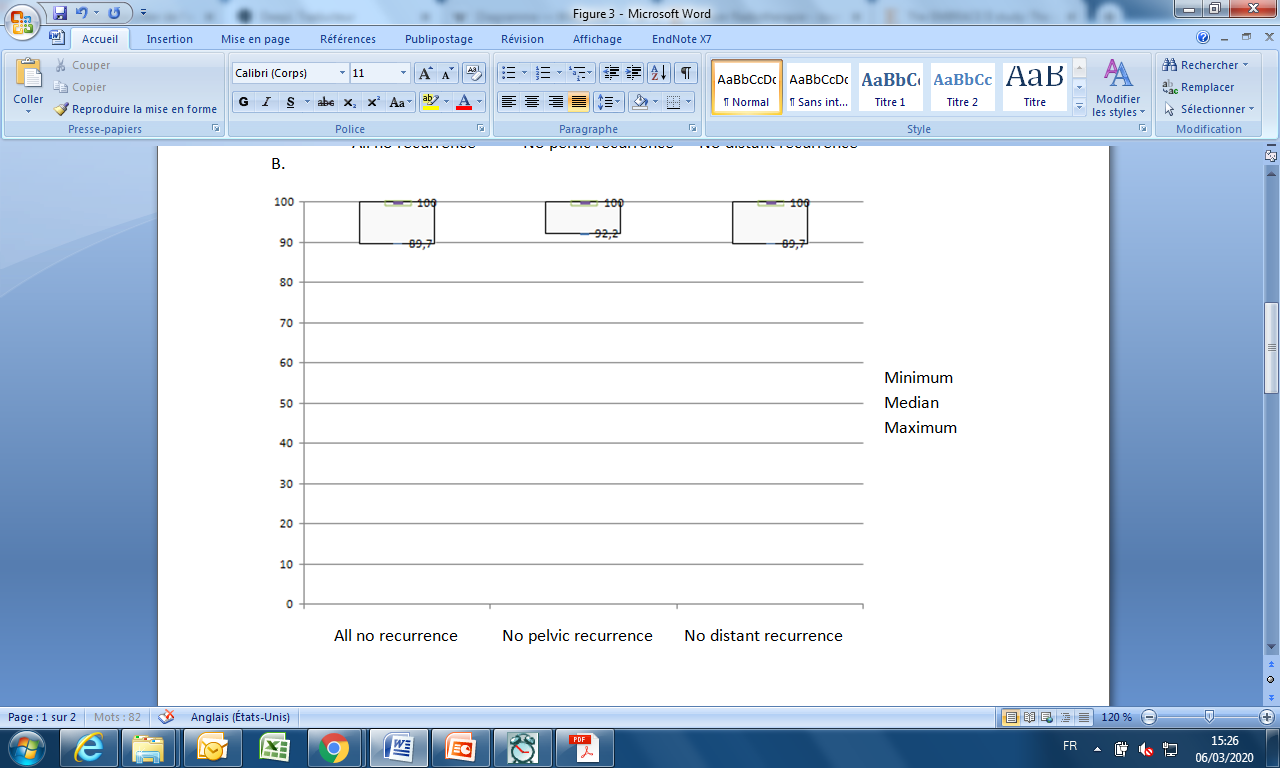


% coverage of the hotspot by isodose 85Gy

Figure s2. Box plots representing the coverage of the hotspot by isodose 80Gy (A) in the recurrence group and (B) in the non recurrence group , there was no difference between 2 groups even in subgroup analysis (p=0.42).

A.


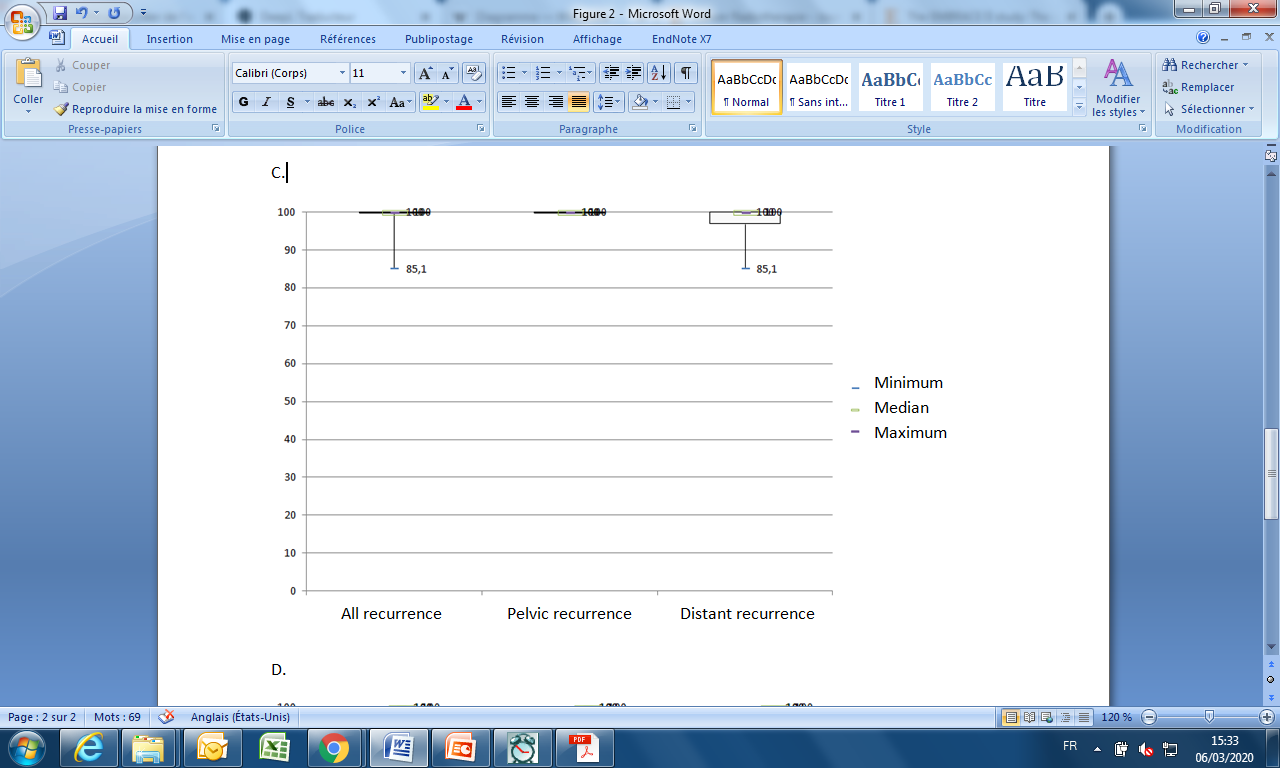


% coverage of the hotspot by isodose 80Gy

B.


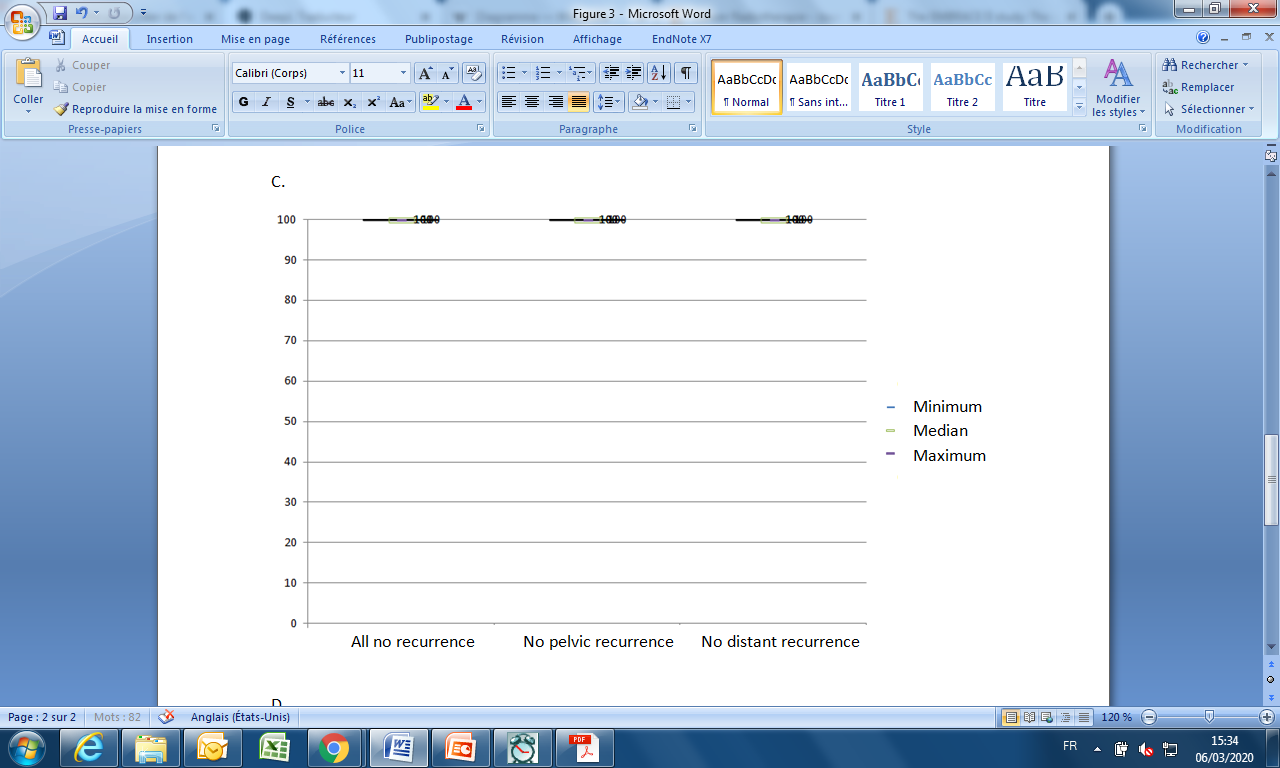


% coverage of the hotspot by isodose 80Gy

Figure s3. Box plots representing the coverage of the hotspot by isodose 78Gy (A) in the recurrence group and (B) in the non recurrence group , there was no significant difference between 2 groups even in subgroup analysis (p=1.00).

A.


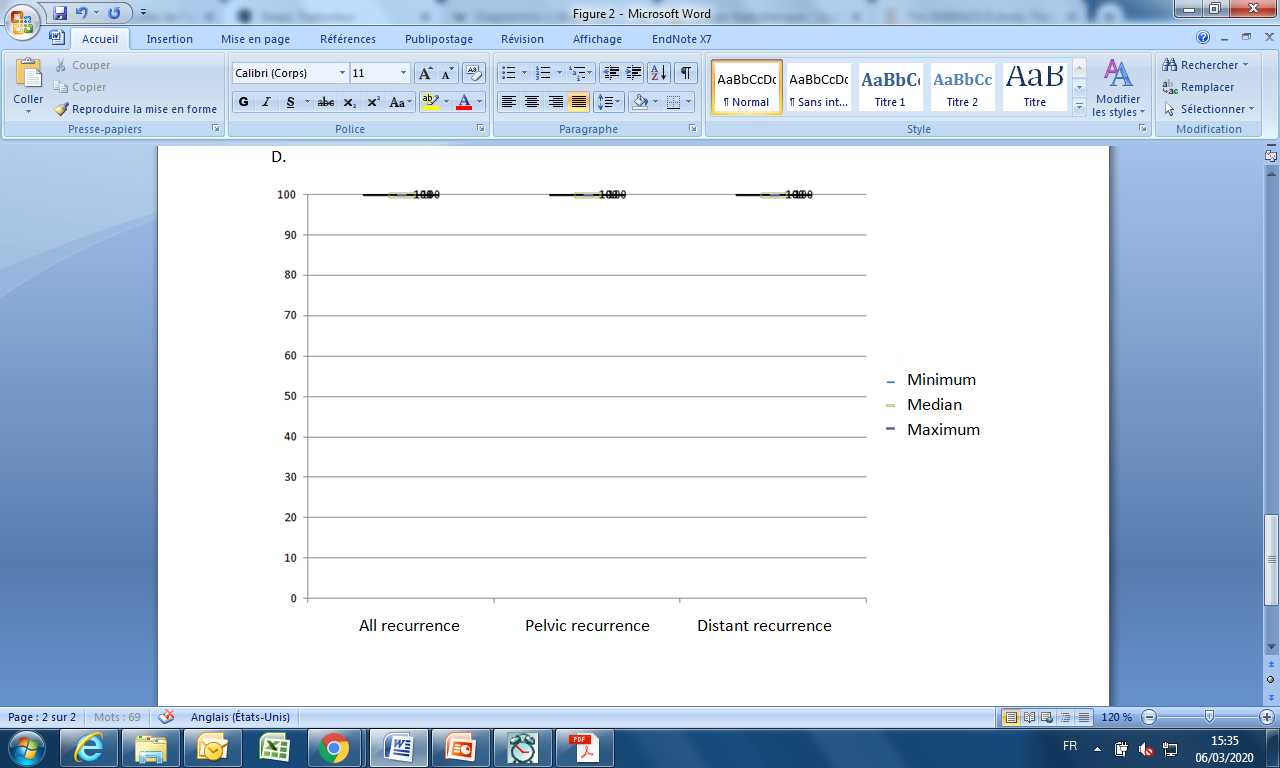


% coverage of the hotspot by isodose 78Gy

B.


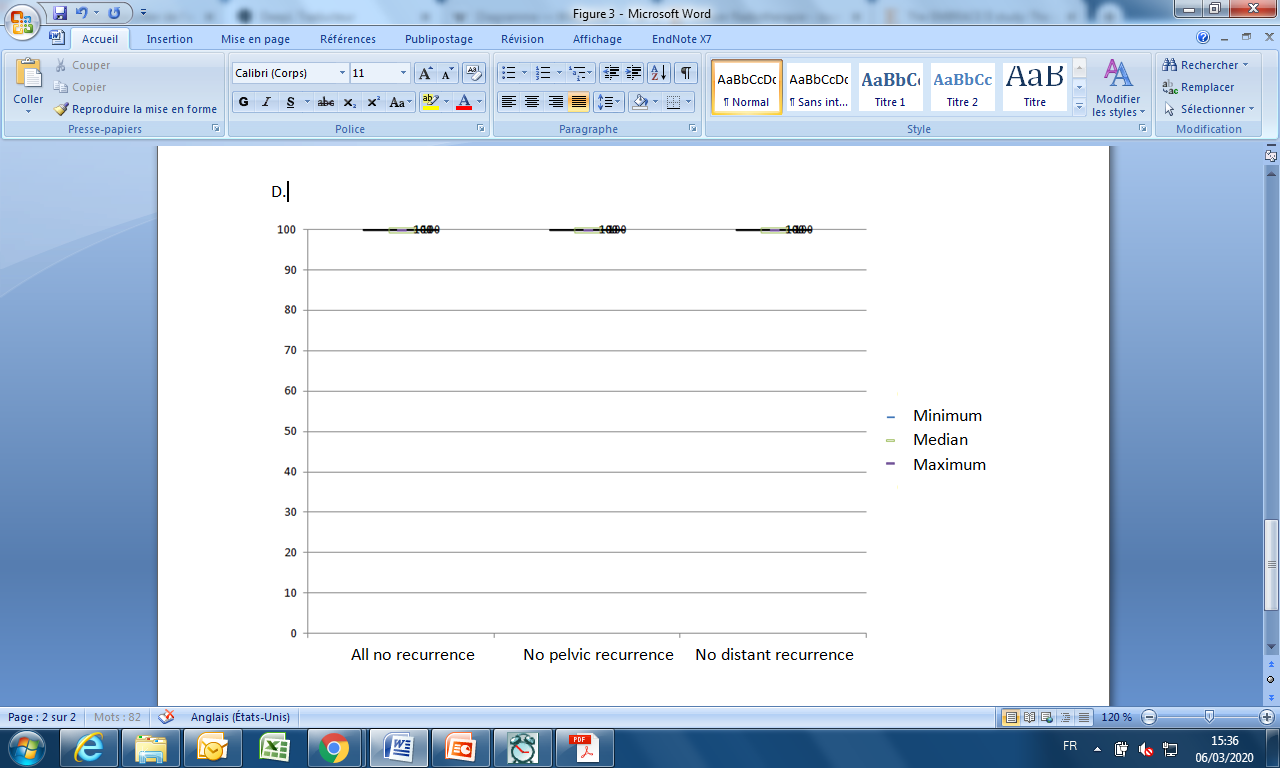


% coverage of the hotspot by isodose 78Gy
